# DAILY MEMORY ISSUES: NATURE, CORRELATES, AND CONSEQUENCES

**DOI:** 10.1093/geroni/igae098.1887

**Published:** 2024-12-31

**Authors:** Johanna Hartung, Gizem Hueluer

**Affiliations:** University of Bonn, Bonn, Nordrhein-Westfalen, Germany; University of Bonn, Bonn, Nordrhein-Westfalen, Germany

## Abstract

Memory lapses are common occurrences in daily life; however, little is known about the mechanisms underlying their occurrence. The overarching goal of this project is to understand the nature, correlates, and consequences of memory lapses in everyday life. We conducted an experience-sampling study over ten days with three measurement occasions per day with 325 adults (age = 18 to 36 years). The momentary assessments included measures of memory lapses, working memory (WM) performance, activities, and mood. Participants also completed a retrospective memory lapse questionnaire. Our results show that the participants who retrospectively reported a higher number of lapses also reported a higher number of lapses across the 30 measurement occasions. Nevertheless, the frequency of momentary memory lapses was unrelated to WM, while the number of retrospectively measured lapses was associated with lower WM. The opposite pattern was found for interference/seriousness: WM was not associated with retrospective ratings of seriousness but with momentary ratings of interference. Memory lapses were more likely to occur on occasions with more cognitive activity and worse mood and individuals who reported more cognitive activity and worse mood than others also reported more memory lapses than others. The occurrence of memory lapses at a given time was unrelated to momentary working memory performance. Taken together, our findings suggest that the occurrence of memory lapses may be indicative of a cognitively active lifestyle, but not necessarily of objective performance deficits. We address the implications of our findings for theory and practice and discuss a replication with older adults.

